# Targeting Kupffer Cell CD44-Mediated Ammonia Death via the ELAVL1-GLS Metabolic Circuit in Radiation-Induced Liver Disease

**DOI:** 10.7150/ijbs.136823

**Published:** 2026-06-17

**Authors:** Yanyan Lin, Shuxuan Wang, Bufu Tang, Pan Zhou, Xiaojun Zhang, Huanliang Chen, Jiaoyang Yang, Shisuo Du, Qian-Qian Zhao, Zhao-Chong Zeng

**Affiliations:** 1 Department of Radiation Oncology, Zhongshan Hospital, Fudan University, Shanghai, 200032, China.; 2 Cancer Center, Zhongshan Hospital, Fudan University, Shanghai, 200032, China.; 3 Department of Interventional Radiology, Zhongshan Hospital, Fudan University, Shanghai, 200032, China.

**Keywords:** radiation-induced liver disease, Kupffer cells, CD44, glutaminase, ammonia-induced cell death

## Abstract

Radiation-induced liver disease (RILD) remains a major dose-limiting toxicity in liver cancer radiotherapy. However, the underlying mechanisms, particularly the immunometabolic reprogramming that sustains inflammatory amplification following irradiation, are still poorly understood. Here, we report that Kupffer cell (KC) depletion significantly attenuated radiation-induced liver inflammation and injury. Irradiation markedly upregulated CD44 expression in KCs, and single-cell RNA sequencing revealed a CD44⁺ macrophage subset associated with injury, inflammatory activation, and metabolic stress signatures. Consistently, genetic deletion or pharmacological inhibition of CD44 protected against RILD by suppressing KC M1 polarization, oxidative stress accumulation, and inflammatory cytokine output. Furthermore, radiation exposure profoundly disrupted nitrogen homeostasis and increased intracellular ammonia levels, effects that were abrogated by CD44 deficiency. Mechanistically, CD44 stabilized the RNA-binding protein ELAVL1 by inhibiting its proteasomal degradation, which subsequently increased the abundance and activity of glutaminase (GLS), elevated the intracellular ammonia burden, and triggered macrophage death. Pharmacological inhibition of GLS with CB-839 rescued the injurious phenotype, while exogenous NH_4_Cl supplementation confirmed the ammonia dependence of the downstream phenotypes. Our findings reveal a CD44-ELAVL1-GLS immunometabolic circuit that links radiation stress to ammonia dysregulation in KCs and subsequent liver injury, suggesting a potential therapeutic target for RILD.

## Introduction

Hepatocellular carcinoma imposes a substantial clinical burden, and many patients present at stages when curative resection or ablation is not feasible^1^-^3^. Liver-directed radiotherapy is increasingly used for liver cancer, particularly for unresectable lesions, to balance local control with functional hepatic reserve^4^-^6^. However, exceeding established thresholds of irradiated liver volume and dose can lead to radiation-induced liver disease (RILD), a pathological process driven by severe oxidative stress and inflammation, which presents as hepatic dysfunction and can progress to liver failure^7^-^9^. The biological programs driving RILD remain incompletely understood, and current clinical management mainly relies on supportive therapy^10^. This diagnostic and therapeutic limitation highlights the urgent need to elucidate core injury mechanisms for developing targeted preventative strategies.

Emerging evidence indicates that RILD is a multicellular process driven not only by parenchymal stress, but also by nonparenchymal cell (NPC) remodeling within the hepatic microenvironment^11^-^13^. Kupffer cells (KCs), the resident macrophages of the liver, constitute a prominent fraction of NPCs and serve as sentinels of tissue homeostasis^14^. Beyond direct target-cell radiotoxicity, the radiation-induced bystander effect propagates sustained sterile inflammation across neighboring tissue zones^15^. Specifically, radiation-induced parenchymal damage triggers massive release of damage-associated molecular patterns (DAMPs) into the extracellular space. Acting as danger signals, these DAMPs are rapidly intercepted by KCs, thereby translating the initial physical injury into an active inflammatory cascade^16^. Consequently, activated KCs rapidly reprogram the local microenvironment through cytokine release and reactive oxygen species (ROS) production^17^, ^18^. However, during RILD, the mechanistic coupling between inflammatory programs and cellular metabolic stress in KCs remains insufficiently defined.

Radiation effects on immune cells can extend beyond DNA damage responses to include metabolic remodeling that reinforces inflammatory activation. In the liver, nitrogen handling is tightly coupled to both tissue homeostasis and immune-cell fitness. Crucially, excessive ammonia accumulation is a potent trigger of mitochondrial dysfunction and oxidative stress in immune cells^19^, ^20^. Recent studies have highlighted the disruption of nitrogen balance as a critical determinant of immune cell survival^21^, ^22^, suggesting that impaired ammonia buffering is an underappreciated contributor to oxidative injury in RILD. Macrophages are particularly vulnerable to metabolic stress because of the absence of a functional urea cycle for enzymatic detoxification. Unlike parenchymal hepatocytes, which possess an immense capacity to clear free ammonia, KCs are intrinsically vulnerable to this nitrogenous stress^23^. Unbuffered free ammonia acts as a lipophilic weak base that readily permeates and alkalinizes highly acidic endolysosomal compartments^24^, ^25^. This rapid neutralization disrupts autophagic flux, triggers mitochondrial permeability transition, and drives an unresolvable collapse of organelle homeostasis, defined as ammonia-induced cell death^26^, ^27^. Paradoxically, this lethal stress is a direct byproduct of immune activation. During M1 polarization, KCs undergo profound metabolic reprogramming to support inflammatory cascades, becoming highly dependent on glutaminolysis as an alternative cellular fuel^28^-^30^. Driven by this metabolic shift, glutaminase (GLS) converts glutamine to glutamate with concomitant release of NH₃, functioning as a major intracellular source of ammonia and a potential regulator of redox balance^31^.

CD44, a hyaluronan receptor best known for its role in adhesion and tumor biology, is widely expressed on myeloid cells and has emerged as a regulator of macrophage activation^32^. Rather than serving solely as a structural anchor, CD44 functions as an advanced microenvironmental sensor, converting extracellular physical stress into profound intracellular remodeling^33^. Recent studies have shown that CD44 can directly bridge these pathological cues to dynamic alterations in mitochondrial metabolism and downstream programs, thereby sustaining inflammatory macrophage phenotypes^34^, ^35^. However, whether CD44 couples radiation stress to nitrogen metabolic imbalance in the liver and whether such an immunometabolic axis contributes to RILD remain unclear. Given these immunometabolic perspectives, we hypothesized that radiation triggers detrimental metabolic reprogramming in KCs driven by CD44 activation, which in turn increases the stability of the ELAVL1-GLS circuit and promotes ammonia accumulation and mitochondrial oxidative injury, ultimately leading to KC death. By identifying the specific CD44-ELAVL1-GLS cascade, our study reveals how highly activated macrophages drive inflammatory amplification during RILD and establishes this pathway as a precise immunometabolic target for mitigating RILD.

## Materials and Methods

### Animals and mouse model construction

Male C57BL/6N mice (6-8 weeks old) were used to establish the RILD model. The mice were anesthetized and subjected to targeted liver irradiation using an Elekta Axesse™ linear accelerator. A single fraction of 25 Gy was delivered to the whole liver (6 MV, 3 Gy/min) to induce acute liver injury, as previously described^36^-^38^, with lead shielding for nonhepatic organs. For KC depletion, clodronate liposomes (Cld-Lipo, 5 mg/ml; Yeasen, China) were administered intraperitoneally (200 μl per mouse) 24 h before treatment. *Cd44* knockout (*Cd44*⁻/⁻) mice on a C57BL/6N background were generated by CRISPR/Cas9 and obtained from Cyagen Biosciences (Suzhou, China). For pharmacological inhibition, a CD44 inhibitor (7 mg/kg, MCE, USA) was administered intraperitoneally starting 24 h before irradiation and continued daily until 48 h after irradiation. All mice were maintained under specific pathogen-free (SPF) conditions, and all animal procedures were approved by the Animal Ethics Committee of Zhongshan Hospital, Fudan University (Approval No. 2023-263).

### Hematoxylin and eosin (HE), immunofluorescence (IF) and immunohistochemistry (IHC) staining

The mice were fasted overnight before the experiment. We first perfused the mouse livers with PBS to remove blood and then fixed them in 4% paraformaldehyde. Clinical specimens were obtained from Zhongshan Hospital with approval from its Institutional Review Board (Approval No. B2025-547). Following paraffin embedding, the tissues were sectioned and subjected to corresponding staining. We then captured images using light or fluorescence microscopy. Antibody information is provided in Supplementary Table S2.

### Flow cytometry

Single-cell suspensions from mouse liver tissues were prepared by perfusion followed by collagenase IV digestion, red blood cell lysis, and filtration. Cultured cells were gently detached, filtered through a 40 μm cell strainer, and collected as single-cell suspensions. Cells were stained with the indicated antibodies or fluorescent probes protected from light and analyzed on a BD FACSAria III flow cytometer. Antibody information is provided in Supplementary Table S3.

### Single-cell RNA sequencing

Following vascular perfusion, mouse liver tissues were immediately collected and dissociated into single-cell suspensions. After filtration and viability assessment, single-cell libraries were generated using the 10× Genomics platform and sequenced by a commercial service provider (OE Biotech, Shanghai, China).

### Statistical analysis

All statistical analyses were performed using GraphPad Prism 9 and R software. Comparisons between two groups were conducted using unpaired two-tailed Student's *t* tests where appropriate. Correlation analyses were performed using Pearson's correlation coefficient, and correlation strength and significance are reported as R and P values. The data are presented as the mean ± SD. Statistical significance was defined as follows: ns, not significant; *P < 0.05, **P < 0.01, ***P < 0.001, and ****P < 0.0001.

## Results

### Radiation induces M1 KC polarization, shaping the inflammatory microenvironment in RILD

**Figure 1:**
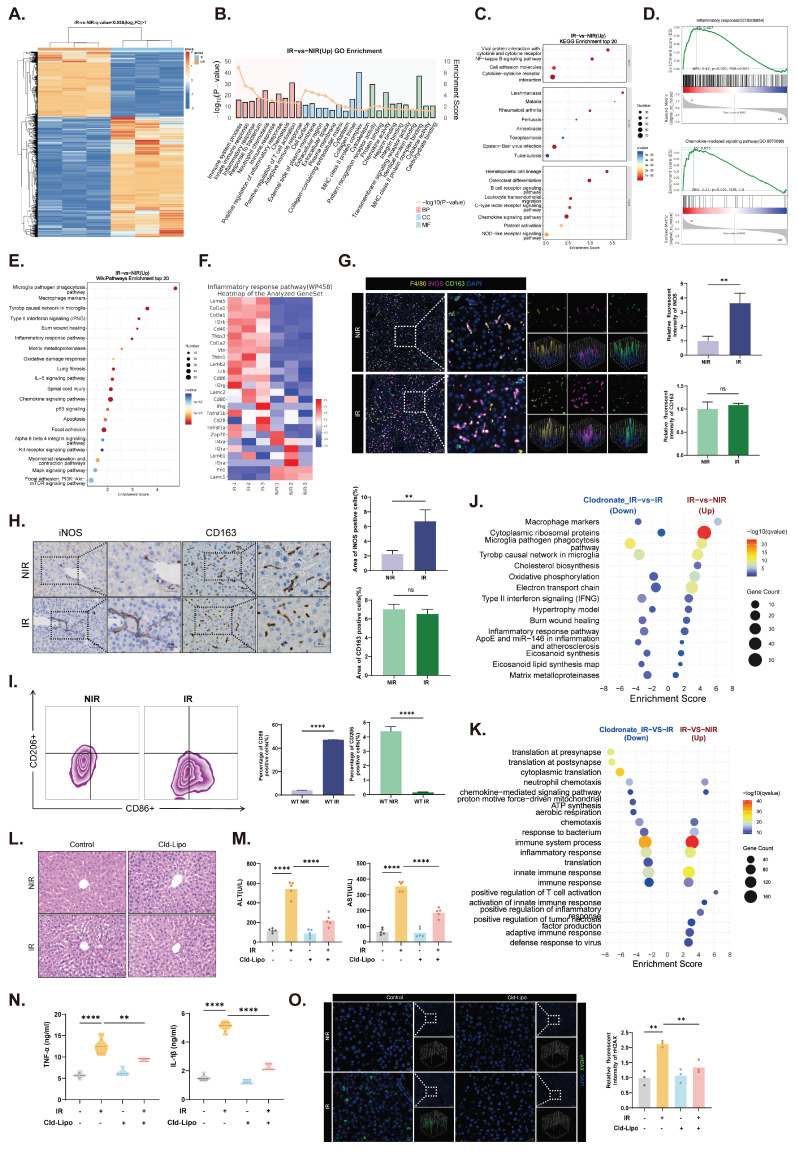
** Irradiation activates KCs toward an M1 phenotype and exacerbates the severity of RILD. (A)** Heatmap showing distinct transcriptional profiles between non-irradiated (NIR) and irradiated (IR) mouse livers. **(B)** GO enrichment analysis of upregulated differentially expressed genes (DEGs) in IR livers (top 30 terms). **(C)** KEGG enrichment of upregulated DEGs in IR group (top 20 pathways). **(D)** GSEA of inflammatory related gene sets. **(E)** WikiPathways enrichment of the top 20 upregulated pathways including macrophage markers. **(F)** Heatmap of representative inflammation-related genes elevated in IR livers. **(G)** IF images of F4/80 (yellow) with iNOS (purple) and CD163 (green) co-staining in the indicated groups. DAPI, blue.** (H)** IHC staining for iNOS and CD163 signals in IR and NIR livers. **(I)** Flow cytometric analysis of CD86⁺ KCs.** (J)** WikiPathways enrichment identifying pathways activated by irradiation and reduced after KCs depletion. **(K)** GO biological process enrichment showing radiation-induced pathways reversed by Cld-Lipo treatment. **(L)** Liver histology changes as shown by HE staining. **(M)** Serum ALT and AST levels (n=5). **(N)** Hepatic TNF-α and IL-1β concentrations (n=5). **(O)** γH2AX staining (green) indicating changes of DNA damage signals. Statistical significance: ns, not significant; **P < 0.01, ****P < 0.0001; two-tailed Student's t-test.

Transcriptomic profiling of liver tissues revealed that irradiation broadly upregulated immune and inflammatory genes (Fig. 1A). Functional analyses indicated strong enrichment of immune activation, chemotaxis, NF-κB signaling, and inflammatory response pathways (Fig. 1B-D). Macrophage activation, including increased expression of M1-associated markers (e.g., *Cd86 and Nos2*) and inflammatory mediators (*Ifng*, *Tnf*, *Il6*, and *Il1b*), was prominent (Fig. 1E and F, S1A and B). Consistent with the transcriptomic changes, KCs adopted an M1-like phenotype after irradiation, as evidenced by increased iNOS levels without a marked change in CD163 levels (Fig. 1G and H). Flow cytometry confirmed a significant increase in M1-like KC polarization after irradiation (Fig. 1I). Concurrently, irradiated KCs exhibited markedly elevated expression of the key recruitment chemokines *Ccl2* and *Cxcl1*, indicating their active role in reshaping the hepatic immune microenvironment (Fig. S1C). These findings indicate that KC M1 polarization is closely associated with the dysregulated inflammatory microenvironment of the irradiated liver.

To determine the functional contribution of KCs, we depleted liver macrophages using Cld-Lipo (Fig. S1D)^39^. Depletion of KCs substantially disrupted the radiation-induced transcriptional program, downregulated the expression of macrophage-related genes, and suppressed the activity of inflammatory pathways (Fig. 1J and K). The pathways attenuated by KC depletion overlapped with those activated by radiation exposure, demonstrating that KCs are essential mediators of the hepatic inflammatory cascade in response to irradiation. Depletion of KCs alleviated radiation-induced liver histologic damage (Fig. 1L). Similarly, the elevations in serum ALT/AST and hepatic TNF-α/IL-1β levels were significantly reduced after KC depletion (Fig. 1M and N). γH2AX staining also revealed markedly reduced DNA damage in irradiated livers after Cld-Lipo treatment, indicating a reduction in radiation-associated genomic injury (Fig. 1O).

Collectively, these data illustrate that radiation-induced inflammation, liver injury, and DNA damage are strongly associated with KCs, indicating that KCs are central drivers of the pathological microenvironment in RILD.

### CD44 is a KC-enriched regulator of RILD pathogenesis

**Figure 2:**
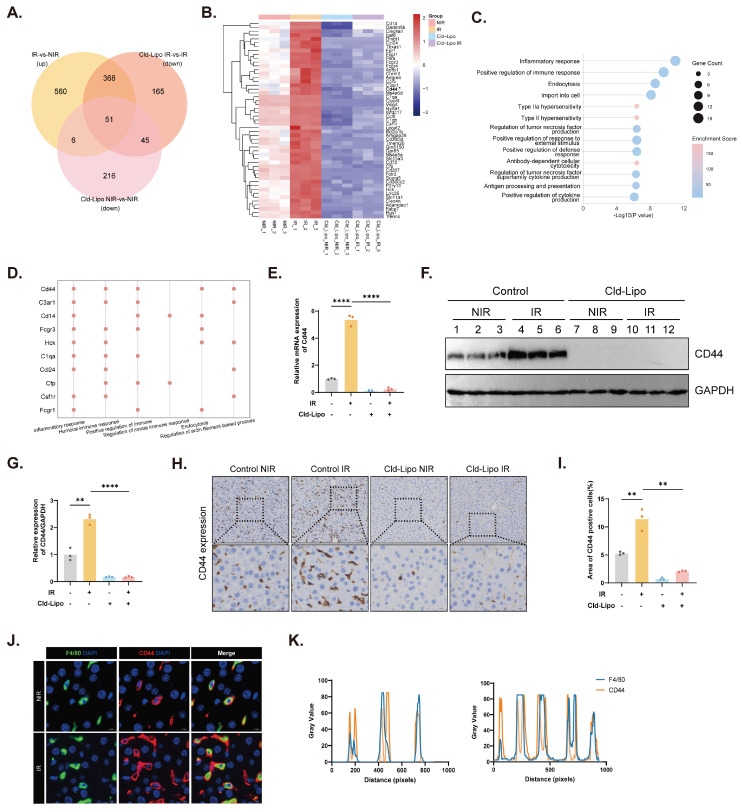
** CD44 defines a potential regulatory node in KCs during RILD. (A)**Venn diagram overlapping 51 candidate genes associated with KC-dependent regulatory node of RILD. **(B)** Heatmap of the 51 candidate genes expression in NIR, IR, Cld-Lipo NIR, and Cld-Lipo IR groups. **(C)** Metascape enrichment analysis of the 51 candidate genes. **(D)** Top 10 immune-associated genes out of the 51 candidates.** (E)** mRNA expression of *Cd44* under the indicated experimental conditions (n=3). **(F** and **G)** Protein expression of CD44 in liver tissues (F) with corresponding quantification (G). **(H** and **I)** CD44 expression in different conditions as shown by IHC staining (H) with corresponding quantification (I) (n=3). **(J** and **K)** IF staining results (J) for CD44 (red) localizing in KCs (F4/80, green) within liver tissues with line scan quantification (K). Statistical significance: **P < 0.01, ****P < 0.0001; two-tailed Student's t-test.

An intersection analysis strategy yielded 51 KC-dependent candidate genes whose expression responded to irradiation but was lost when KCs were removed (Fig. 2A and B). Metascape enrichment analysis revealed that these genes were predominantly involved in immune and inflammatory pathways (Fig. 2C). Among the top-ranked candidates, *Cd44* emerged together with *C3ar1*, *Cd14*, *Fcgr3*, *Hck*, *C1qa*, *Ccl24*, *Cfp*, *Csf1r*, and *Fcgr1* (Fig. 2D). We confirmed marked CD44 upregulation at both the RNA and protein levels after irradiation, whereas this was largely abolished by KC depletion (Fig. 2E-G, S2A). IHC staining further confirmed these results (Fig. 2H and I), and co-immunofluorescence localized CD44 to F4/80⁺ macrophages (Fig. 2J and K). These findings establish CD44 as a KC-enriched mediator activated during RILD.

**Figure 3:**
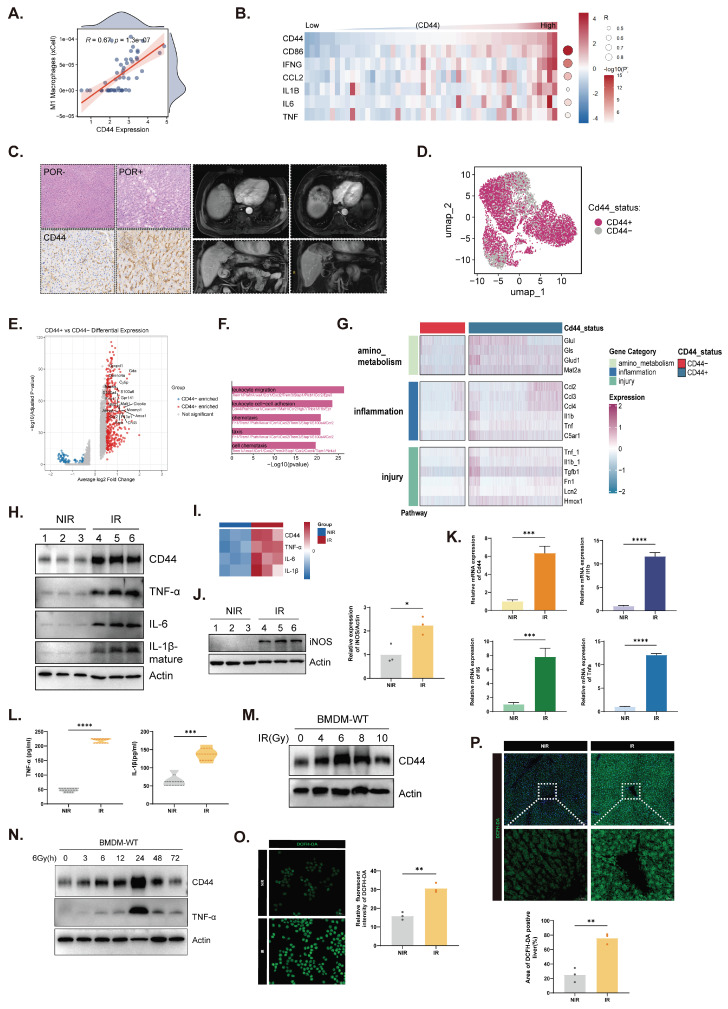
** CD44 drives M1 macrophage polarization after radiation. (A)** Analysis of normal liver tissue from TCGA between *CD44* expression and the M1 macrophage gene signature. **(B)** Correlation heatmap of positive relationships between *CD44* and representative M1 markers and inflammatory cytokines including *CD86, IFNG, CCL2, IL1B, IL6, TNF* from TCGA normal liver tissues. **(C)** Representative samples from RILD patients' livers before (POR-) and after (POR+) radiotherapy, showing HE staining (top panel), IHC staining of CD44 (bottom panel), and MRI images (right panel). **(D)** UMAP visualization of single-cell RNA sequencing data of macrophage subpopulations in mouse livers based on CD44 expression. **(E)** DEG analysis between CD44⁺ and CD44⁻ macrophages. **(F)** GO analysis revealed CD44⁺ macrophages were specifically associated with immune-related pathways. **(G)** Pathway enrichment analysis in CD44⁺ macrophages. **(H**, **I** and** K)** Expression of CD44 and pro-inflammatory cytokines in KCs under indicated treatment. **(J)** Protein expression of iNOS in KCs with quantification (n=3).** (L)** Levels of cytokines in KCs conditioned media (n=5). **(M** and** N)** Expression of CD44 in BMDMs in response to radiation dose (M) and over time (N). **(O** and** P)** ROS levels in BMDMs (O) and liver tissue (P) detected by DCFH-DA with corresponding quantification. *P < 0.05, **P < 0.01, ***P < 0.001, ****P < 0.0001; two-tailed Student's t-test.

Analysis of normal liver samples from the TCGA cohort revealed a significant positive correlation between *CD44* expression and an aggregate M1 macrophage signature (Fig. 3A). Consistently, correlation heatmaps further demonstrated that *CD44* expression was positively associated with the expression of individual M1 markers and inflammatory mediators, including *CD86*, *IFNG*, *CCL2*, *IL1B*, *IL6*, and *TNF* (Fig. 3B). The clinical relevance of CD44 was supported by paired human liver specimens collected before and after radiotherapy. Postirradiation samples displayed characteristic pathological features of RILD together with pronounced CD44 upregulation in irradiated regions (Fig. 3C). Radiologic assessment revealed MRI features characteristic of RILD, including a T1-hypointense hepatic lesion with peripheral enhancement, heterogeneous internal signals, and accompanying perfusion abnormalities (Fig. 3C).

Single-cell RNA sequencing of mouse livers further distinguished CD44⁺ from CD44⁻ macrophage subpopulations, revealing distinct transcriptional signatures (Fig. 3D and E). CD44⁺ macrophages were enriched in inflammatory, injury, and amino acid metabolic pathways (Fig. 3F and G), indicating the emergence of a specialized proinflammatory subset during RILD. In KCs, irradiation increased the expression of CD44 and the proinflammatory cytokines TNF-α, IL-6, and IL-1β at the mRNA and protein levels (Fig. 3H, I, and K), as well as the M1 marker iNOS (Fig. 3J). These mediators were also elevated in KC-conditioned media (Fig. 3L). In bone marrow-derived macrophages (BMDMs), irradiation also induced CD44 expression, which peaked at 6 Gy and 24 h (Fig. 3M and N). Increased ROS production in irradiated BMDMs and livers reflected heightened oxidative stress, consistent with these activated states (Fig. 3O and P). Cell-cell communication analysis further mapped the extensive ligand-receptor interactions between this CD44⁺ subset and NPCs, highlighting its core role in orchestrating immune remodeling (Fig. S3A-C). Together, these findings support CD44 as a radiation-response node associated with proinflammatory macrophage polarization in RILD.

### CD44 deficiency protects against RILD by suppressing KC M1 polarization

**Figure 4:**
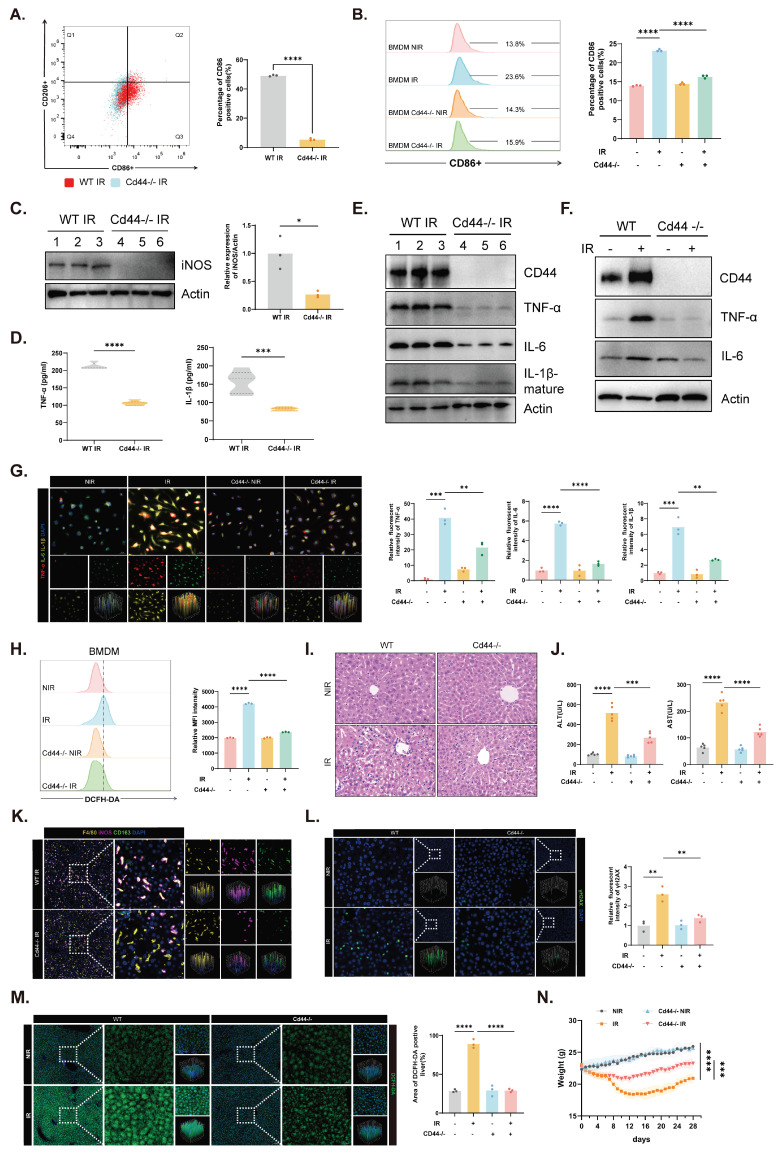
** Effects of CD44 deficiency on liver injury and macrophage activation following irradiation. (A** and **B)** CD86⁺ cells in KCs (A) and BMDMs (B) under different conditions with representative images (left) and quantification (right) (n=3). **(C)** Immunoblot analysis of iNOS in KCs isolated from WT and *Cd44*⁻/⁻ mice under irradiation (left) and corresponding quantification (right). **(D)** Cytokine concentrations in conditioned media from KCs (n=5). **(E** and **F)** Expression of CD44 and cytokines in KCs and BMDMs. **(G)** IF images (left) of inflammatory mediator expression in BMDMs across the indicated groups with corresponding quantification (right). **(H)** ROS levels in BMDMs. **(I)** Representative liver histology from WT and *Cd44*⁻/⁻ mice under NIR and IR conditions. **(J)** Serum ALT and AST levels in mice with or without irradiation (n=5). **(K)** IF staining of iNOS, CD163, and F4/80 in mice liver sections under IR conditions. **(L)** γH2AX staining in liver sections in the four groups. **(M)** ROS levels in liver tissues under NIR and IR conditions (left) with corresponding quantification (right). **(N)** Body-weight changes of mice before and after irradiation (n=5). *P < 0.05, **P < 0.01, ***P < 0.001, ****P < 0.0001; two-tailed Student's t-test.

*Cd44*⁻/⁻ mice were constructed to evaluate the functional contribution of CD44 (Fig. S4A). Under physiological conditions, CD44 deficiency did not cause obvious histological abnormalities in major organs (Fig. S4B). Compared with those in control mice, M1 polarization was suppressed in both KCs and BMDMs derived from *Cd44*⁻/⁻ mice (Fig. 4A-C), indicating that CD44-dependent macrophage activation was attenuated. Furthermore, conditioned media from irradiated *Cd44*⁻/⁻ KCs contained lower concentrations of secreted inflammatory mediators (Fig. 4D). *Cd44*⁻/⁻ KCs also presented reduced TNF-α, IL-6, and IL-1β expression at both the protein and mRNA levels (Fig. 4E, S4C and D). A diminished inflammatory response was similarly observed in *Cd44*⁻/⁻ BMDMs, as confirmed by immunoblot analyses and IF (Fig. 4F and G). Compared with control cells, ROS formation in irradiated *Cd44*⁻/⁻ BMDMs was reduced (Fig. 4H). Moreover, the suppression of M1 polarization in *Cd44*⁻/⁻ KCs was accompanied by reduced expression of *Ccl2* and *Cxcl1* (Fig. S4E), further supporting the role of CD44 in KC-driven immune remodeling.

*In vivo*, compared with control mice, irradiated *Cd44*⁻/⁻ mice developed less liver injury, with improved histology and serum ALT/AST levels (Fig. 4I and J). Lower iNOS expression was observed in liver sections from irradiated *Cd44*⁻/⁻ mice than in those from control mice (Fig. 4K and S4F). The percentage of γH2AX-positive cells was also reduced in irradiated *Cd44*⁻/⁻ livers, indicating less DNA damage (Fig. 4L). Furthermore, less ROS formation in irradiated *Cd44*⁻/⁻ liver tissues was consistent with the cellular phenomenon (Fig. 4M). Concurrently, CD44 deficiency alleviated overall body weight loss and promoted faster recovery during the postirradiation period (Fig. 4N and S4G).

These data collectively reveal that CD44 is required for the irradiation-induced M1 polarization of KCs and that CD44 deficiency broadly protects against RILD by restraining macrophage-driven inflammatory signaling and oxidative stress.

### CD44 regulates ammonia dysregulation and macrophage death following irradiation

**Figure 5:**
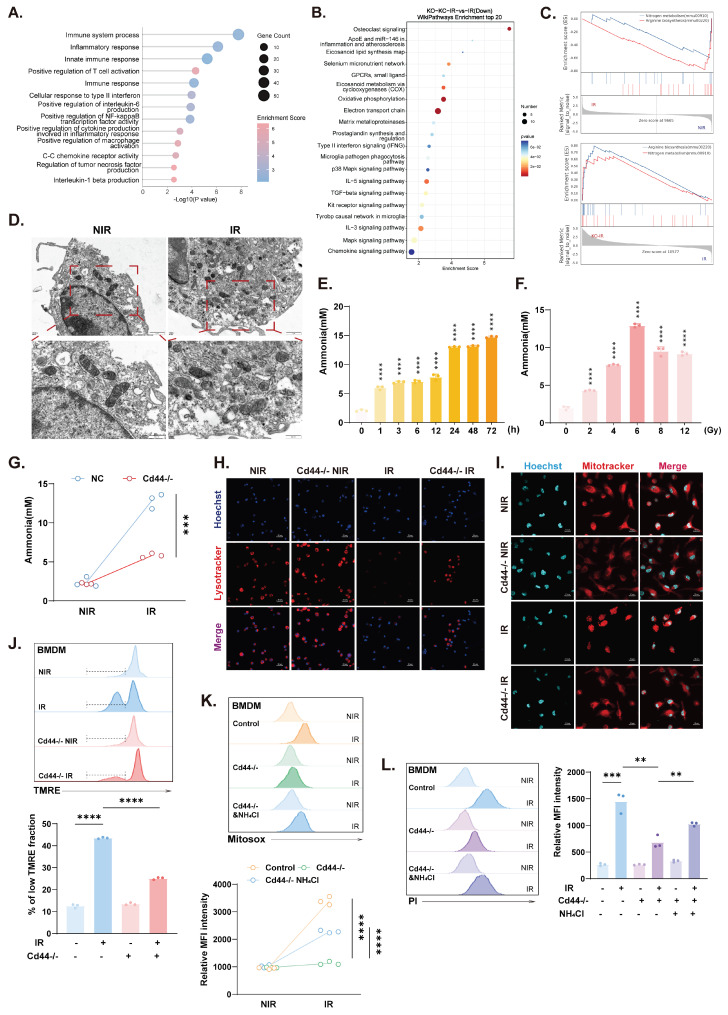
** CD44 links irradiation to ammonia metabolic dysregulation and macrophage death. (A** and **B)** GO biological process and KEGG pathway enrichment analysis of genes downregulated in *Cd44*⁻/⁻ KCs compared to WT KCs under irradiation.** (C)** GSEA results showing suppression of arginine and nitrogen metabolism in IR KCs compared with NIR KCs, with restoration upon *Cd44*⁻/⁻ under IR conditions. **(D)** TEM images of mitochondrial structural damage in BMDMs following IR treatment. **(E** and** F)** Time- and dose-dependent changes in intracellular ammonia levels in BMDMs following irradiation. **(G)** Intracellular ammonia levels in BMDMs. **(H** and **I)** Lysosomal (H) and mitochondrial (I) signals in BMDMs. **(J)** Flow cytometric analysis of mitochondrial membrane potential (top), followed by quantification (bottom). **(K)** Mitochondrial ROS levels of BMDMs, with or without NH_4_Cl (12 mM, 24 h). Representative histograms, top; quantification, bottom. **(L)** Macrophage death under NIR or IR conditions in BMDMs, with or without NH_4_Cl. Histograms, left; quantification, right. Statistical significance: **P < 0.01, ***P < 0.001, ****P < 0.0001; n=3; two-tailed Student's t-test.

To define the downstream mechanisms of CD44 in RILD, we performed transcriptomic profiling of KCs isolated from WT and *Cd44*⁻/⁻ mice. Functional enrichment revealed attenuated inflammatory, immune, and oxidative stress pathways in irradiated *Cd44*⁻/⁻ KCs compared with controls (Fig. 5A and B). GSEA further revealed broad metabolic perturbations in irradiated KCs, including changes in nitrogen metabolism, amino acid metabolism, the urea cycle, and arginine metabolism (Fig. 5C, S5A). In particular, primary KCs exhibited minimal expression of core urea cycle enzymes (*Cps1*, *Otc*,* Ass1*, and *Asl*) compared with hepatocytes (Fig. S5B-E), indicating a limited intrinsic capacity for ammonia clearance. Further analysis at the single-cell level revealed that a glutamine/ammonia-stress module was predominantly enriched within the CD44⁺ KC subpopulation (Fig. S5F). Notably, these metabolic disturbances were largely reversed by CD44 deficiency (Fig. 5C, S5A), suggesting that irradiation reprograms KC metabolism toward a CD44-dependent nitrogen-stress state, setting the stage for impaired ammonia homeostasis.

Transmission electron microscopy (TEM) revealed mitochondrial swelling and cristae disruption in irradiated BMDMs (Fig. 5D), which is consistent with the ultrastructural features of ammonia-induced cell death^21^. Intracellular ammonia levels increased in irradiated BMDMs in a time- and dose-dependent manner (Fig. 5E and F), and this effect was markedly reversed in *Cd44*⁻/⁻ cells (Fig. 5G), indicating that CD44-dependent disruption of ammonia metabolism occurred. Crucially, direct measurement of hepatic ammonia confirmed that the irradiation-induced ammonia burden was significantly attenuated in *Cd44*⁻/⁻ mice compared with controls (Fig. S5G), consistent with the protection observed *in vitro*. Furthermore, ammonia dysregulation in irradiated macrophages was accompanied by lysosomal impairment and mitochondrial defects, including structural disruption, depolarization, and oxidative stress (Fig. 5H-K). Depletion of CD44 significantly alleviated this multi-organelle stress and protected macrophages from ammonia-induced cell death (Fig. 5L). To assess the causal role of ammonia toxicity, exogenous NH_4_Cl was applied at 12 mM for 24 h to recapitulate the pathological ammonia accumulation and stress observed in our irradiated BMDMs. The addition of NH_4_Cl alone had a minimal effect, while it abolished the protection conferred by CD44 deficiency, restoring mitochondrial ROS levels and cell death to those observed in the irradiated controls (Fig. 5K and L).

As a result, irradiation disrupts ammonia homeostasis in macrophages in a CD44-dependent manner. CD44 depletion partially restored ammonia metabolic balance, attenuated organelle damage, and protected macrophages from radiation-induced death, indicating that the a CD44-ammonia axis is involved in this process.

### CD44 engages an ELAVL1-GLS circuit to drive ammonia-dependent macrophage dysfunction

**Figure 6:**
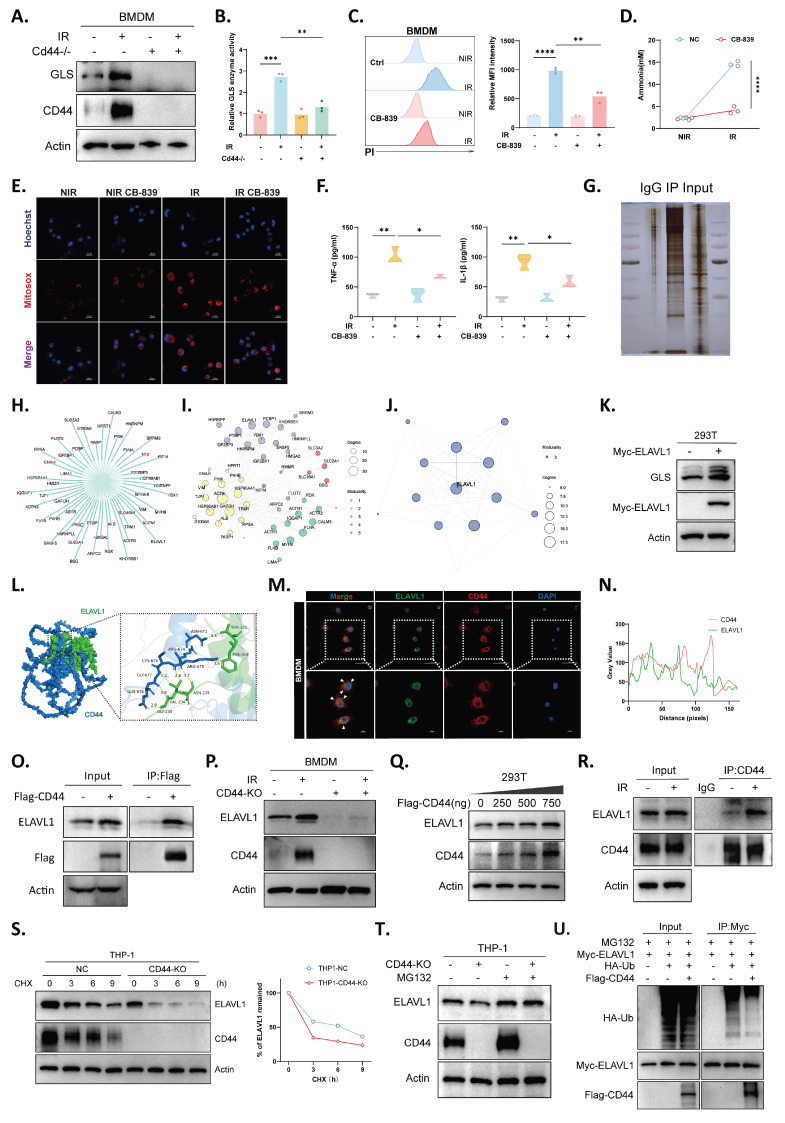
** CD44 stabilizes ELAVL1 to enhance GLS-dependent metabolic dysfunction in macrophages. (A)** Immunoblot analysis of GLS and CD44 with or without irradiation. **(B)** GLS enzymatic activity measured in BMDMs under the indicated conditions. **(C)** Propidium iodide staining showing macrophage death in response to irradiation and GLS inhibitor CB-839; representative histograms (left) and quantification (right). **(D)** Measurement of intracellular ammonia levels in BMDMs under irradiation with or without CB-839 (n=3). **(E)** Mitochondrial ROS levels in BMDMs following irradiation and GLS inhibition (CB-839, 5 μM). **(F)** Levels of TNF-α and IL-1β concentration of BMDM-conditioned media following irradiation and GLS inhibition (n=3).** (G)** Silver staining gel images of immunoprecipitated complexes from CD44-overexpressing 293T cells. **(H)** PPI network constructed from the intersection of CD44 pull-down candidates and STRING-annotated CD44 interactors. **(I)** Three major functional modules identified by cluster analysis of the PPI network.** (J)** Hub analysis highlighting ELAVL1 as a top-ranked candidate within the network. **(K)** Protein expression of GLS and MYC-ELAVL1 overexpression in 293T cells. **(L)** Molecular docking model of the CD44-ELAVL1 interaction. Docking score: -268.83. **(M** and **N)** IF images (M) showing co-localization of CD44 (red) and ELAVL1 (green) in macrophages with corresponding line scan quantification (N). **(O)** Coimmunoprecipitation in 293T cells with or without Flag-CD44 overexpression. **(P)** Protein expression of CD44 and ELAVL1 under NIR or IR conditions. **(Q)** Immunoblot analysis showing dose-dependent induction of ELAVL1 and CD44 in 293T cells. **(R)** Increased CD44-ELAVL1 association under IR condition in 293T cells endogenous coimmunoprecipitation. **(S)** ELAVL1 protein stability measured by cycloheximide (CHX, 100 µg/mL) chase assay in THP-1 macrophages. **(T)** Protein expression of ELAVL1 in THP-1 with or without treatment of MG132 (10 μM). **(U)** Reduced polyubiquitination of ELAVL1 with Flag-CD44 overexpression. Statistical significance: *P < 0.05, **P < 0.01, ***P < 0.001, ****P < 0.0001.

We next examined whether CD44 regulates GLS, the major ammonia-producing enzyme. In BMDMs, irradiation clearly increased GLS expression and enzymatic activity, which were markedly attenuated in *Cd44*⁻/⁻ cells (Fig. 6A and B). Pharmacological inhibition of GLS with CB-839^40^ reduced macrophage death (Fig. 6C), decreased ammonia accumulation (Fig. 6D), alleviated mitochondrial superoxide stress (Fig. 6E), and suppressed inflammatory cytokine release under irradiation (Fig. 6F), indicating that GLS-dependent ammonia production occurs downstream of CD44.

An LC-MS interactome screen in CD44-overexpressing cells identified a set of candidates linking CD44 to GLS regulation (Fig. 6G). A protein-protein interaction (PPI) network integrating these candidates with STRING-predicted interactors was constructed (Fig. 6H) and organized into several distinct functional modules (Fig. 6I), with hub analysis highlighting ELAV-like RNA binding protein 1 (ELAVL1/HuR) as a central node (Fig. 6J). Since ELAVL1 is known to stabilize GLS mRNA and increase GLS expression^41^, we considered the CD44-ELAVL1-GLS axis as a potential mechanism linking CD44 to ammonia production. Indeed, ELAVL1 overexpression increased GLS protein levels (Fig. 6K). Structural modeling revealed a binding interface between CD44 and ELAVL1 (Fig. 6L). Consistent with this prediction, confocal imaging revealed their intracellular colocalization (Fig. 6M and N), and their direct interaction was confirmed by coimmunoprecipitation (Fig. 6O). In macrophages, irradiation drove parallel upregulation of both proteins, whereas CD44 deficiency blunted the induction of ELAVL1 expression (Fig. 6P), supporting the role of ELAVL1 as a downstream component regulated by CD44.

Mechanistically, increasing CD44 expression increased ELAVL1 protein level in a dose-dependent manner without altering *ELAVL1* mRNA level (Fig. 6Q, S6A). Radiation also strengthened the endogenous interaction between CD44 and ELAVL1 (Fig. 6R). Protein stability assays revealed accelerated ELAVL1 degradation upon CD44 loss (Fig. 6S), whereas proteasome inhibition restored ELAVL1 levels in *CD44*⁻/⁻ cells (Fig. 6T). Moreover, ubiquitination assays revealed reduced ELAVL1 polyubiquitination in the presence of CD44 overexpression (Fig. 6U), indicating that CD44-dependent regulation of ELAVL1 stability occurs via the ubiquitin-proteasome pathway. Together, these data support a mechanistic cascade in which CD44 stabilizes ELAVL1, thereby enhancing GLS-driven ammonia production and promoting macrophage dysfunction under irradiation. Notably, this coordinate upregulation of ELAVL1 and GLS and its dependence on CD44 were also observed *in vivo*, as irradiated whole-liver lysates from *Cd44*⁻/⁻ mice showed markedly reduced ELAVL1 and GLS protein levels compared with controls (Fig. S6B). Analysis of public liver transcriptomic cohorts further revealed a positive correlation between CD44 and GLS expression (Fig. S6C), underscoring the translational value of this immunometabolic axis.

### Targeting CD44 alleviates RILD by restoring inflammatory and metabolic homeostasis

**Figure 7:**
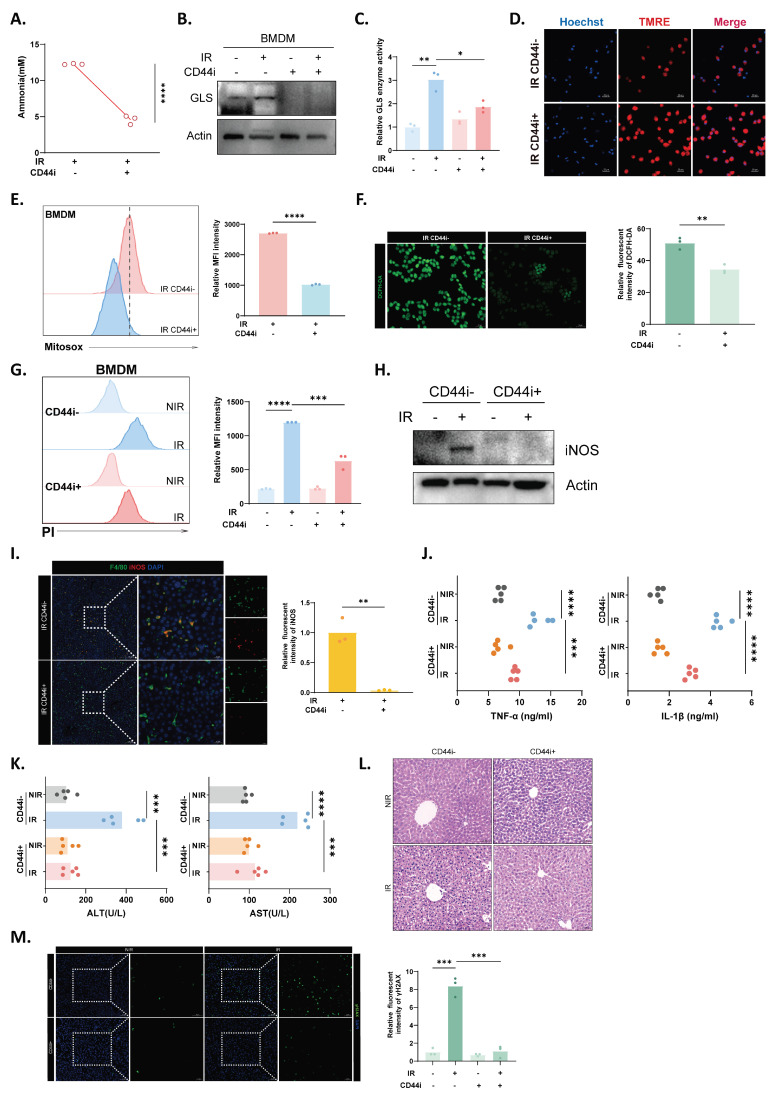
** Inhibition of CD44 protects against RILD through the inflammatory-metabolic axis. (A)** Intracellular ammonia levels in BMDMs treated with or without CD44 inhibitor (CD44i) following irradiation (n=3). **(B)** Protein expression of GLS in BMDMs under indicated conditions. **(C)** Enzymatic activity of GLS in BMDM lysates following various treatment (n=3).** (D)** Mitochondrial membrane potential assessed by TMRE staining. **(E)** Mitochondrial superoxide levels presented as histograms (left) and quantification (right) (n=3). **(F)** Measurement of total cellular ROS levels in BMDMs (left), with quantification (right) (n=3). **(G)** Propidium iodide staining showing cell death rate, shown as representative histograms (left) and quantification (right) (n=3). **(H)** Protein expression levels of iNOS in BMDMs under CD44i- and CD44i+.** (I)** iNOS (red) expression in liver tissues (left) with relative quantification (right) (n=3).** (J)** Hepatic concentrations of TNF-α and IL-1β (n=5). **(K)** Serum ALT and AST levels across groups (n=5).** (L)** Histology changes of liver sections with or without CD44i treatment.** (M)** DNA damage in liver sections assessed by γH2AX (green) IF staining (left) and corresponding quantification (right) (n=3). Statistical significance: *P < 0.05, **P < 0.01, ***P < 0.001, ****P < 0.0001; two-tailed Student's t-test.

CD44 was targeted with a hyaluronic acid-based inhibitor both *in vitro* and *in vivo*^42^. Pharmacological inhibition of CD44 significantly suppressed intracellular ammonia accumulation, along with radiation-induced GLS expression and enzymatic activity in macrophages (Fig. 7A-C). This metabolic restoration coincided with improved mitochondrial membrane integrity (Fig. 7D), a marked reduction in both mitochondrial and cellular ROS generation (Fig. 7E and F), and a subsequent decrease in macrophage death (Fig. 7G). Furthermore, CD44 blockade effectively restrained the proinflammatory response, as evidenced by downregulated iNOS expression in both macrophages and liver tissues (Fig. 7H and I) and reduced hepatic TNF-α and IL-1β levels (Fig. 7J). Consequently, this intervention significantly decreased serum ALT and AST levels (Fig. 7K), which is consistent with diminished inflammatory infiltration and preserved tissue architecture upon HE staining (Fig. 7L). At the molecular level, CD44 inhibitor-treated livers exhibited mitigated DNA damage, as indicated by a substantially diminished γH2AX-positive area (Fig. 7M).

In summary, our findings suggest that targeting CD44 exerts a protective effect against RILD through the suppression of inflammatory activation in macrophages and the restoration of metabolic homeostasis. These results provide preclinical evidence that CD44 is a potential therapeutic target for RILD.

## Discussion

RILD represents a complex pathology driven by sustained oxidative stress and inflammatory propagation, which limits the efficacy of liver-directed radiotherapy^7^, ^43^. While physical dose constraints are currently the primary preventive measure, they fail to address the underlying molecular and metabolic drivers of injury^44^. Although inflammatory signaling and microenvironmental reprogramming are increasingly recognized as integral to RILD pathobiology, the dominant cellular sources of ROS and metabolic checkpoints that enhance this oxidative burst remain incompletely defined^45^-^47^. Evaluating the molecular programs that connect radiation exposure to immunometabolic stress and inflammatory amplification therefore remains a critical need.

A central contribution of this study is the establishment of a conceptual framework linking radiation-induced oxidative stress to immunometabolic reprogramming in RILD. Within this framework, we identify KCs not only as immune responders but also as the primary drivers of redox imbalance and inflammatory amplification. We defined CD44 as a key driver linking radiation exposure to metabolic dysfunction in KCs. As guardians of hepatic innate immunity, KCs swiftly recognize injury signals and initiate inflammatory cascades that influence hepatic homeostasis and damage pathways^48^, ^49^. This study connects KC-driven inflammatory amplification to a defined and pharmacologically tractable molecular axis, providing a mechanistic basis for therapeutic targeting in RILD.

CD44 is best known as an adhesion and migration receptor involved in tumor stemness and therapeutic resistance^50^, but accumulating evidence supports its active regulatory functions in macrophages rather than serving as a passive marker. Emerging studies support a pathogenic contribution of myeloid CD44 to hepatic inflammation and injury through the modulation of local stress environments^51^, ^52^. However, whether CD44 plays a KC-centered role in RILD and how it relates radiation stress to metabolic stress and inflammatory amplification have not been defined. Our study addresses this gap by placing CD44 upstream of a mechanistically actionable KC immunometabolic cascade.

**Figure 8:**
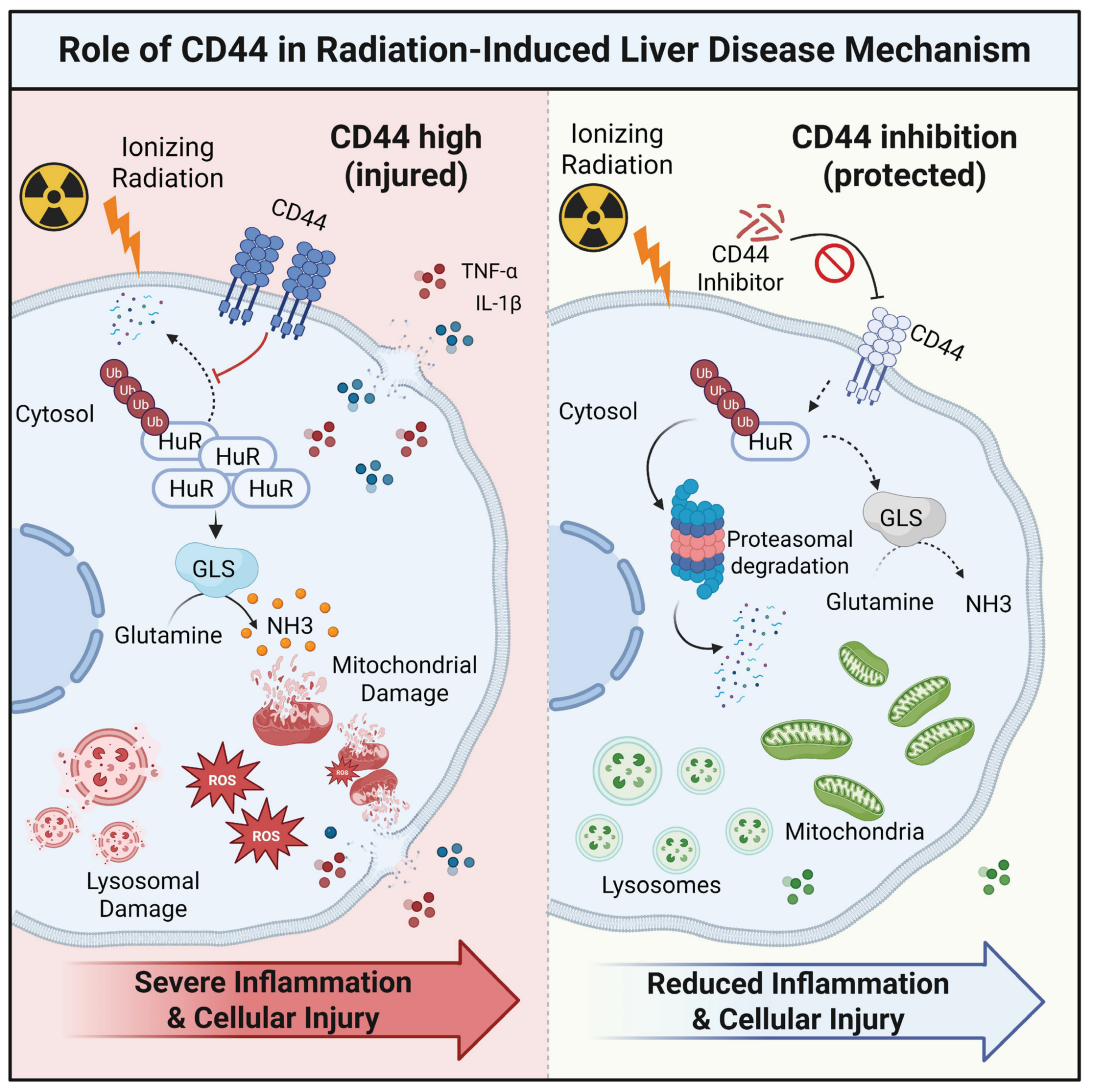
** A working model depicting CD44 as a central node driving KCs cell dysfunction, linking metabolic stress to inflammatory signaling in RILD**.

In accordance with this hypothesis, CD44 expression was strongly induced in irradiated livers and isolated KCs, which is consistent with the upregulation of oxidative stress markers and M1 polarization. Mechanistically, CD44 functions upstream of an immunometabolic switch by stabilizing ELAVL1/HuR, thereby increasing GLS activity and driving excessive ammonia accumulation. The resulting toxic ammonia burden induces mitochondrial impairment, amplifies inflammation, and leads to ammonia-induced macrophage death, thereby worsening RILD (Fig. 8). While our ubiquitination data clearly reveal that CD44 protects ELAVL1 from proteasomal degradation, the precise molecular dynamics governing this process remain unclear. We speculate that the intracellular domain of CD44 may physically interact with ELAVL1 to induce steric hindrance, thereby shielding critical lysine residues from specific E3 ubiquitin ligases known to target ELAVL1, such as β-TrCP1^53^. Alternatively, CD44 might act through competitive inhibition by sequestering the responsible E3 ligase or indirectly by recruiting specific deubiquitinases such as USP20^54^ to dynamically reverse ELAVL1 ubiquitination. Collectively, these findings suggest that CD44 is not merely a passive biomarker but a decisive node that couples radiation stress to metabolic and inflammatory dysfunction in KCs, linking macrophage failure to tissue-level hepatic injury. Beyond post-transcriptional regulation, the upstream transcriptional circuitry directing the inflammatory CD44⁺ KC state remains to be identified. Preliminary regulon analysis of our single-cell data suggests that transcription factors linked to myeloid activation and stress responses, including* Nfil3*, *Klf4*, *Cebpb*, and* Stat2*, are selectively active in CD44⁺ KCs. Elucidating how these factors intersect with the CD44-ELAVL1-GLS axis will further clarify the initiation of immunometabolic amplification during RILD.

Ammonia-induced cell death is a recently described immune cell fate linked to ammonia imbalance and has rarely been considered in the context of RILD^21^, ^27^, ^55^. Our data underscore that radiation injury in macrophages extends beyond canonical inflammatory signaling or DNA damage. Radiation also perturbs metabolic homeostasis, promotes toxic ammonia accumulation, and induces organelle failure. This provides a mechanistic route through which metabolic and oxidative stress intensifies inflammatory escalation, sustaining a feedforward loop within the irradiated liver microenvironment. Both genetic and pharmacological inhibition of CD44 led to improved ammonia homeostasis, decreased oxidative inflammatory burden, and reduced liver tissue injury. Moreover, NH_4_Cl add-back and CB-839 experiments revealed ammonia handling downstream of CD44 and upstream of mitochondrial dysfunction, supporting a model in which irradiation activates CD44 to disrupt ammonia homeostasis in KCs and thereby create a self-reinforcing inflammatory state during RILD. Pharmacologically targeting this glutaminolysis checkpoint is a compelling dual therapeutic opportunity, particularly considering the established clinical profile of CB-839 in advanced oncology trials. Nevertheless, the ubiquitous expression of CD44 across primary tissues raises valid concerns regarding systemic off-target toxicity. Future therapeutic avenues must therefore prioritize localized delivery strategies. Employing vehicles targeted specifically to the liver, such as peptide-functionalized nanocarriers or engineered liposomes tailored for selective uptake by KCs^56^-^58^, represents an optimal path to harness this protective mechanism without compromising systemic safety. By establishing dysregulated ammonia metabolism and GLS activity as core components of KC pathology, we expand the mechanistic landscape of RILD toward the immunometabolic dimension.

In conclusion, we identified a CD44-ELAVL1-GLS axis that links radiation stress to abnormal ammonia accumulation, mitochondrial collapse, and oxidative stress in KCs. This metabolic program leads to ammonia-induced cell death, thereby amplifying inflammatory liver injury. These findings expand the immunometabolic landscape of RILD and provide a tractable mechanistic foundation for KC-focused preventive strategies. Importantly, CD44 provides a molecular entry point for preventive intervention, complementing current physical dose constraints. It remains to be determined whether ammonia dysregulation extends to other hepatic immune subsets and how KCs coordinate with hepatocytes and stellate cells within a spatially organized injury niche. Apart from normal tissue injury, this conserved immunometabolic axis likely operates within tumor-associated macrophages in irradiated hepatocellular carcinoma. Unlike resident KCs, which experience fatal ammonia toxicity, tumor-associated macrophages might divert glutaminolysis products for survival while utilizing secreted ammonia to suppress infiltrating cytotoxic T lymphocytes. Although the specific metabolic adaptations in these complex microenvironments warrant further investigation, strategically combining targeted CD44 or GLS inhibitors with localized radiotherapy is poised to offer a dual benefit, simultaneously protecting normal liver tissue and creating a more favorable immunological context for tumor control. Additionally, comprehensive preclinical evaluation and prospective clinical studies are needed to define safety, efficacy, and patient selection.

## Supplementary Material

Supplementary methods, figures and tables.

## Data Availability

All data are available in the main text, the supplementary materials, and from the corresponding author upon reasonable request.
