# Supplementary material for: Targeting Kupffer Cell CD44-Mediated Ammonia Death via the ELAVL1-GLS Metabolic Circuit in Radiation-Induced Liver Disease
Source: Int J Biol Sci. 2026 Jun 17;22(12):6417–33. doi: 10.7150/ijbs.136823 (PMC13412084; doi:10.7150/ijbs.136823)
Supplement: Supplementary file 1 — Supplementary methods, figures and tables. [file ijbsv22p6417s1.pdf]

This PDF file includes:

Supplementary Materials

Supplementary Figure S1 to S6.

Supplementary Table S1 to S3.

Supplementary materials

RNA isolation and RT-qPCR

Cellular RNA was extracted using RNA lysis buffer. RNA samples were either submitted for RNA sequencing (OE Biotech, Shanghai, China) or purified using a commercial RNA extraction kit (Yeasen) according to the manufacturer's instructions. Complementary DNA (cDNA) was synthesized using a reverse transcription kit (Yeasen). Quantitative real-time PCR was performed using SYBR Green Master Mix (Yeasen). Relative gene expression levels were calculated using the $2^{-\Delta\Delta Ct}$ method and normalized to GAPDH. Primer sequences used for RT–qPCR are listed in the Supplementary Table S1.

ELISA assay

Levels of TNF-α and IL-1β were measured using commercially mouse TNF-α and mouse IL-1β high-sensitivity ELISA kit (Abclonal, China) according to the manufacturers' instructions. Liver tissues were processed to obtain tissue interstitial fluid by mincing the tissues on ice, followed by PBS washing and sequential centrifugation at 1,000 × g for 3 min, 2,000 × g for 8 min, and 15,000 × g for 30 min at 4°C. The final supernatants were collected for cytokine measurement. For cell culture samples, conditioned media were cleared by centrifugation at 1,000 × g for 10 min prior to detection.

Western blotting

Cells were lysed in RIPA buffer supplemented with protease and phosphatase inhibitors on ice. Protein lysates were mixed with loading buffer, denatured, followed by separation via SDS–PAGE.The separated proteins were then transferred onto a PVDF membrane. After transfer, membranes were blocked with 5% nonfat milk and incubated with primary antibodies at 4 °C overnight, followed by HRP-conjugated secondary antibodies at room temperature. Protein bands were visualized using the Tanon imaging system and quantified using ImageJ software. Antibody information is provided in the Supplementary Table S2.

Intracellular ROS detection

Mice were fasted for 24 h before tissue collection. Fresh liver tissues were rapidly harvested, snap-frozen, and processed for cryosectioning. Cultured cells were analyzed under live-cell conditions. Intracellular ROS levels were detected using the DCFH-DA fluorescent probe (KeyGEN BioTECH, China) according to the manufacturer's instructions, followed by analysis using fluorescence microscopy or flow cytometry.

Primary cell isolation and culture

We isolated KCs from mouse livers using a two-step collagenase perfusion method. After anesthesia, the portal vein of mice was cannulated for in situ perfusion. We first perfused livers with calcium- and magnesium-free D-Hanks' solution to remove blood, following with collagenase IV–containing digestion buffer until tissue softening. The liver was excised and further digested briefly, and enzymatic activity was terminated with serum-containing RPMI 1640 medium. The cell suspension was filtered and centrifuged at low speed to obtain hepatocytes. NPCs were collected from the supernatant and subjected to density gradient centrifugation using a 30%–60% Percoll gradient. Cells at the interface were collected as KC–enriched fractions, resuspended in RPMI 1640 supplemented with 10% fetal bovine serum, and

plated for 1 h to allow adherence. Non-adherent cells were removed, and adherent cells were used as KCs.

BMDMs were generated from mouse bone marrow cells following red blood cell lysis and filtration. Cells were cultured in macrophage differentiation medium supplemented with M-CSF (40 ng/mL). Fresh medium was added on day 3, and mature macrophages were obtained after 7 days of differentiation.

HEK 293T cells were maintained in DMEM supplemented with 10% fetal bovine serum and 1% penicillin–streptomycin. THP-1 cells were cultured in RPMI 1640 with the same supplements and differentiated into macrophage-like cells by treatment with phorbol 12-myristate 13-acetate (PMA, 50 ng/mL) for 48 h. All cells were maintained at 37 °C in a humidified incubator with 5% CO_2.

TEM assay

Cells were fixed with pre-cooled electron microscopy fixative (ASPEN, America) and processed for TEM using standard procedures. Ultrathin sections were examined with a transmission electron microscope (Tecnai G2 20 TWIN, China), with a focus on mitochondrial ultrastructural features.

Ammonia concentration measurement

Cells were collected, washed with PBS, and resuspended in assay buffer prior to lysis by sonication on ice. Lysates were clarified by centrifugation, and supernatants were subjected to ammonia measurement using an Ammonia Assay Kit (Sigma-Aldrich, MAK310) according to the manufacturer’s instructions. Absorbance was measured using a SpectraMax iD3 microplate reader (Molecular Devices).

Mitochondrial and lysosomal function assays

Live cells were subjected to the indicated treatments and incubated separately with mitochondrial or lysosomal fluorescent probes, including MitoSOX (MCE), TMRE (MCE), Mito-Tracker (Beyotime), and Lyso-Tracker (Beyotime), according to the manufacturers' instructions. After staining, cells were washed and counterstained with Hoechst (Beyotime) as indicated. Fluorescence signals were acquired using confocal microscopy (FV3000RS) or quantified by flow cytometry.

GLS activity assay

GLS activity was measured using a commercial assay kit (Elabscience) according to the manufacturer's instructions. BMDMs were collected after the indicated treatments, lysed by sonication on ice, and subjected to enzymatic and colorimetric reactions. Absorbance was measured using a microplate reader, and relative GLS activity was calculated accordingly.

Immunoprecipitation

HEK 293T cells were transfected with the indicated plasmids and subjected to different treatments. Cells were lysed in RIPA buffer supplemented with protease and phosphatase inhibitors on ice. Clarified lysates were incubated either with the indicated antibodies followed by capture with protein A/G magnetic beads, or directly with tag-conjugated magnetic beads at 4°C overnight. Immunocomplexes were washed, eluted, and analyzed by western blotting. Antibody information is provided in the Supplementary Table S2.

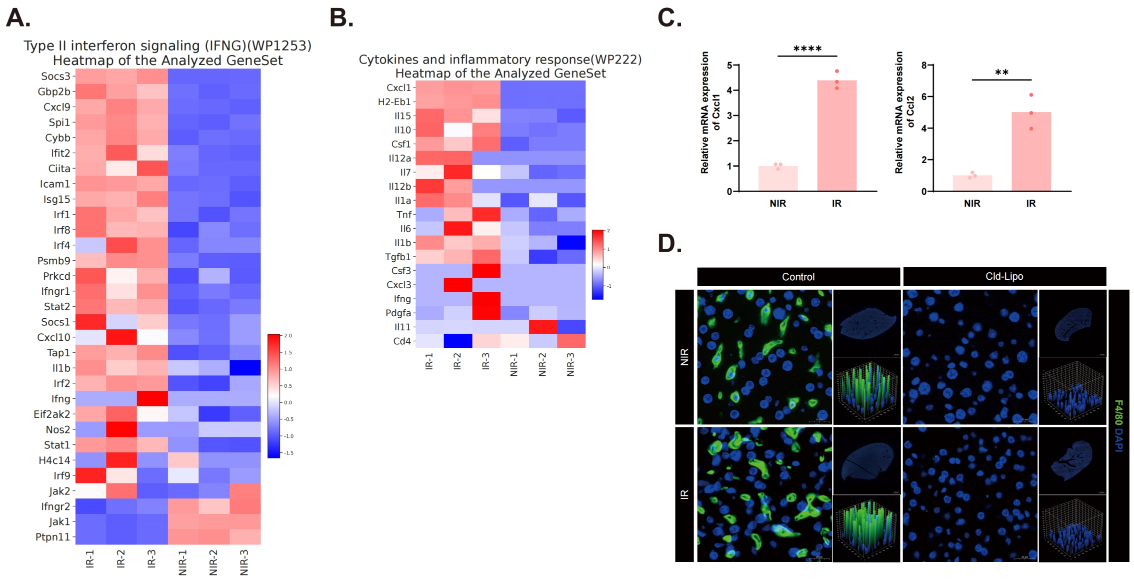

Fig. S1. Irradiation activates inflammatory signaling, which is effectively reversed by Cld-Lipo treatment. (A and **B)** Heatmap of representative inflammation-related genes elevated in IR livers. **(C)** mRNA expression of *Cxcl1* and *Ccl2* in KCs (n=3). **(D)** IF staining of F4/80 demonstrating effective macrophage depletion following Cld-Lipo treatment. F4/80, green; DAPI, blue. Statistical significance: **P < 0.01, ****P < 0.0001.

A.

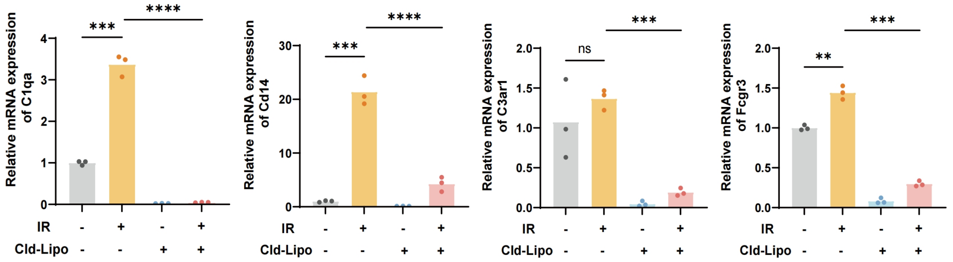

Fig. S2. RILD-related genes expression. (A) mRNA expression of selected candidate genes under the indicated experimental conditions (n=3). Statistical significance: ns, not significant; **P < 0.01, ***P < 0.001, ****P < 0.0001.

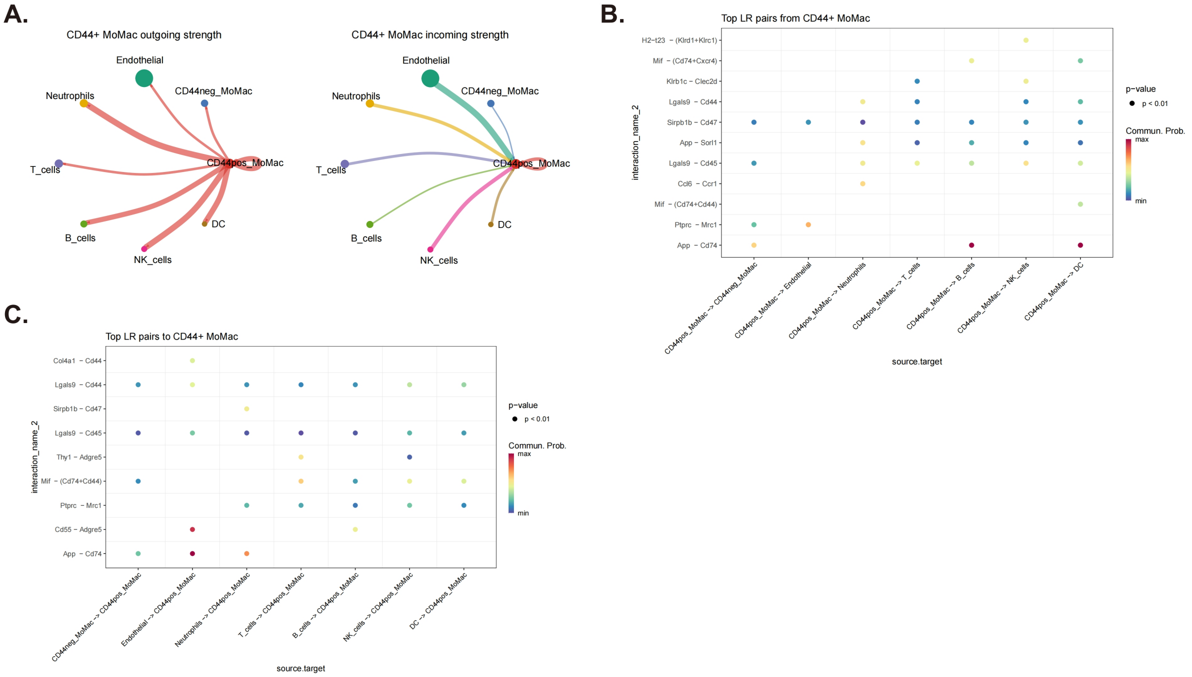

Fig. S3. Ligand-receptor interactions centered on the CD44^{+} macrophage subset. (A) Circle plots depicting the overall signaling strength between CD44^{+} macrophages and other NPCs. Left: outgoing signals from CD44^{+} macrophages. Right: incoming signals to CD44^{+} macrophages. **(B** and **C)** Bubble plots of the top ligand-receptor pairs used by CD44^{+} macrophages as signal senders (B) or signal receivers (C).

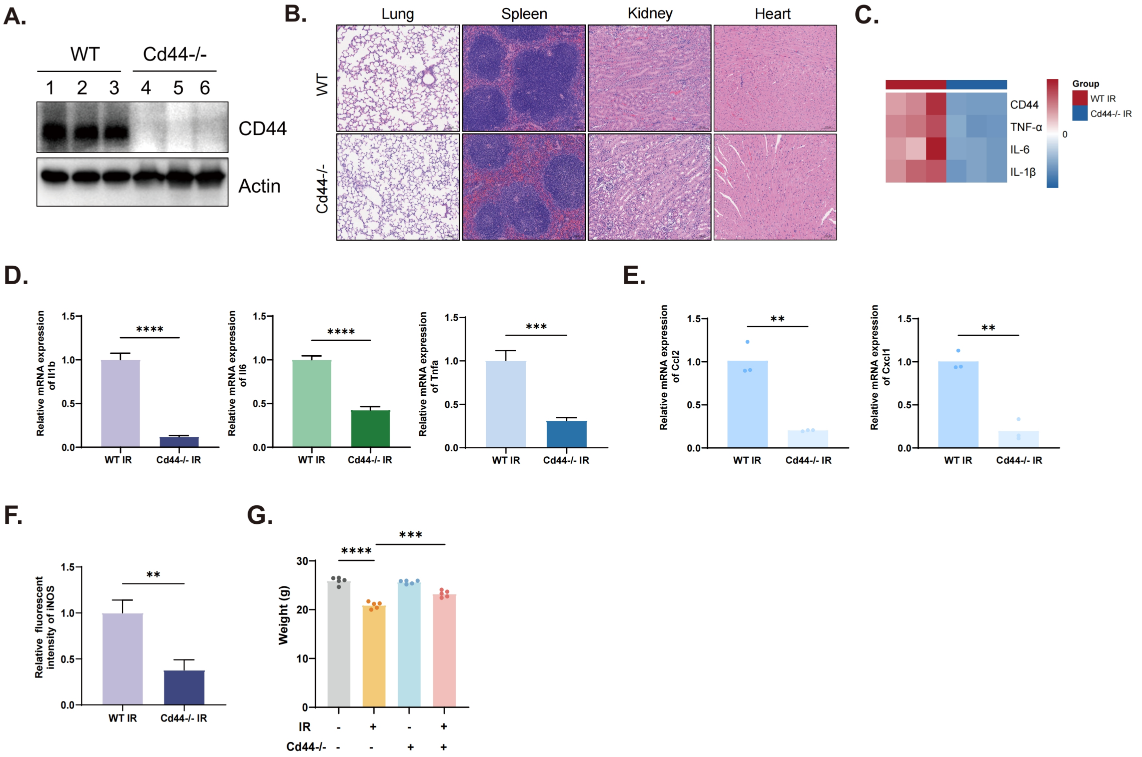

Fig. S4. Deficient of CD44 mitigates the liver inflammation and injury under irradiation. **(A)** Immunoblot analysis confirming the absence of CD44 protein in liver tissue from *Cd44*⁻/⁻ mice. **(B)** Histological evaluation of lung, spleen, kidney, and heart from WT and *Cd44*⁻/⁻ mice under baseline conditions. **(C)** Heatmap of densitometric values derived from immunoblot analyses of KCs. **(D)** mRNA expression of *Il1b*, *Il6*, and *Tnfa* in KCs from the indicated groups. **(E)** mRNA expression of *Ccl2*, and *Cxcl1* in KCs under irradiation. **(F)** Quantification of iNOS positive cells in mice liver sections under IR conditions (n=3). **(G)** Quantification assessment of body weight in WT and *Cd44*⁻/⁻ mice under NIR and IR conditions (n=5). Statistical significance: **P < 0.01, ***P < 0.001, ****P < 0.0001.

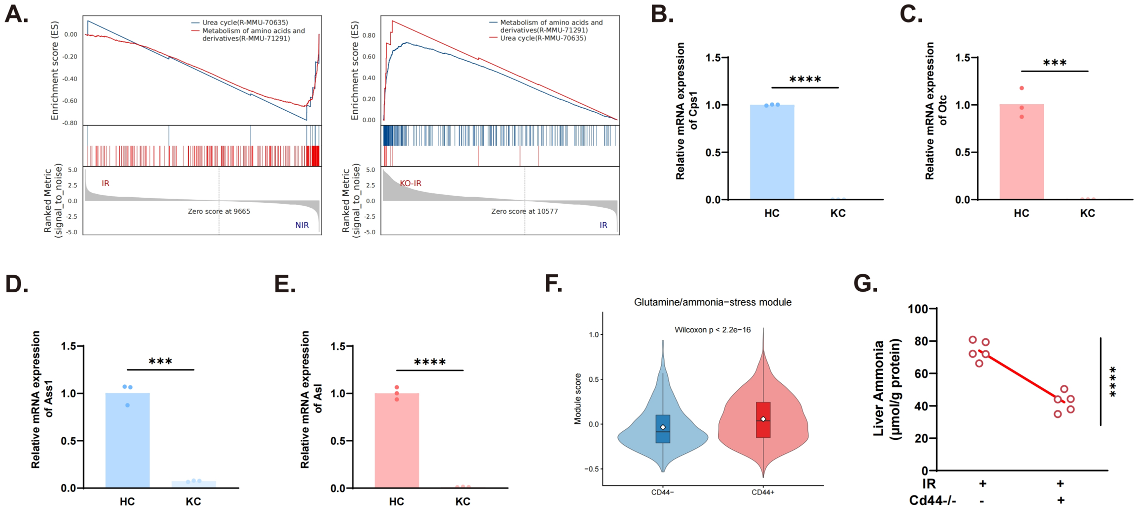

Fig. S5. Radiation triggers ammonia-associated immunometabolic dysfunction in KCs. (A) GSEA plots showing coordinated alterations in urea cycle and amino acid metabolism pathways in IR versus *Cd44*-KO-IR groups. **(B** to **E)** qPCR analysis of the urea cycle enzymes *Cps1* (B), *Otc* (C), *Ass1* (D), and *Asl* (E) in primary HC and KC (n=3). **(F)** Violin plot showing the enrichment score of glutamine/ammonia-stress gene module in CD44^{+} versus CD44^{-} KCs from scRNA-seq data. **(G)** Ammonia concentration in liver homogenates from IR WT and *Cd44*$^{-/-}$ mice (n=5). Statistical significance: ***P < 0.001, ****P < 0.0001.

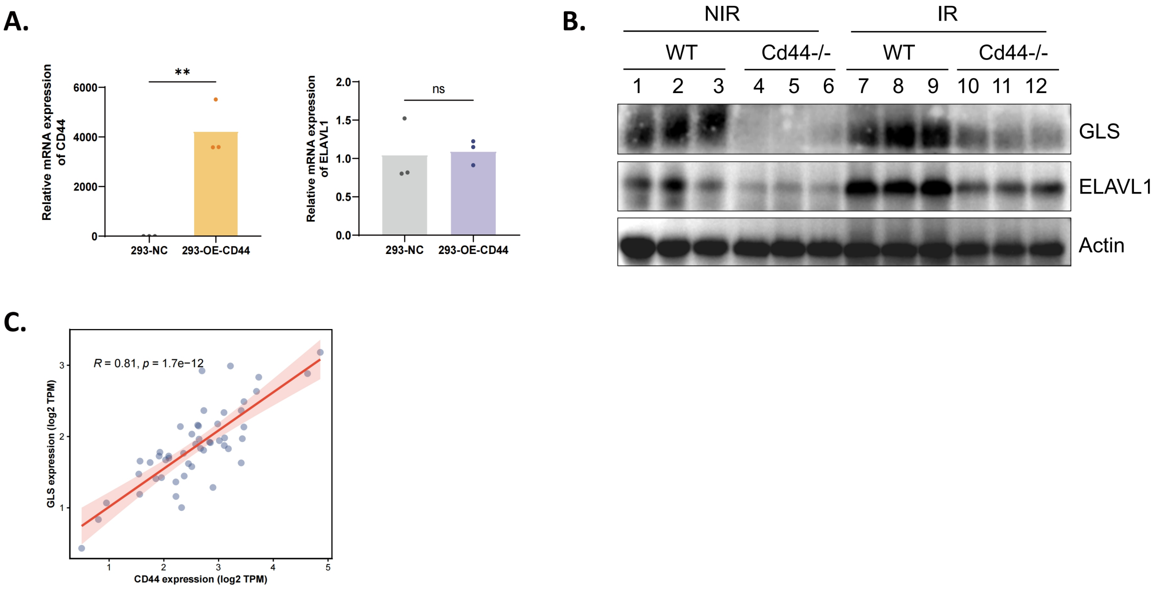

Fig. S6. *In vivo* and clinical validation of the CD44-ELAVL1-GLS axis. (A) Quantitative RT–PCR analysis showing increased *CD44* mRNA expression while *ELAVL1* mRNA levels remain unchanged. **(B)** ELAVL1 and GLS protein levels in whole-liver lysates from IR WT and *Cd44*$^{-/-}$ mice. **(C)** Correlation analysis of *CD44* and *GLS* mRNA expression in adjacent normal liver samples from the TCGA-LIHC dataset. Statistical significance: ns, not significant; $^{**}P < 0.01$.

Table S1. Primer sequence of RT-qPCR.

Primer	Sequence
mCd44-F	CCACAGCCTCCTTTCAATAACC
mCd44-R	GGAGTCTTCGCTTGGGGTA
mGapdh-F	AGGTCGGTGTGAACGGATTTG
mGapdh-R	TGTAGACCATGTAGTTGAGGTCA
mC3ar1-F	TCGATGCTGACACCAATTCAA
mC3ar1-R	TCCCAATAGACAAGTGAGACCAA
mCd14-F	CTCTGTCCTTAAAGCGGCTTAC
mCd14-R	GTTGCGGAGGTTCAAGATGTT
mC1qa-F	TTCGGCAGAACCCAATGACG
mC1qa-R	TGGTATGGACTCTCCTGGTTG
mFcgr3-F	GGTACCACACTGCTTTCTCCCT
mFcgr3-R	ACTTCCTCCAGTAATCCCTCGG
mTnfa-F	CAGGCGGTGCCTATGTCTC
mTnfa-R	CGATCACCCCGAAGTTCAGTAG
mIl6-F	GAGGATACCACTCCCAACAGACC
mIl6-R	AAGTGCATCATCGTTGTTCATACA
mIl1b-F	GCAACTGTTCCTGAACTCAACT
mIl1b-R	ATCTTTTGGGGTCCGTCAACT
hCD44-F	CTGCCGCTTTGCAGGTGTA
hCD44-R	CATTGTGGGCAAGGTGCTATT
hELAVL1-F	AACTACGTGACCGCGAAGG
hELAVL1-R	CGCCCAAACCGAGAGAACA
hGAPDH-F	GGAGCGAGATCCCTCCAAAAT
hGAPDH-R	GGCTGTTGTCATACTTCTCATGG

Table S2. Antibodies for Western blot, IF and IHC.

<table>
<tr><td>Name</td><td>Dilution</td><td>Species</td><td>Manufacture</td><td>Cat, No.</td><td>Application</td></tr>
<tr><td>F4/80</td><td>1:100</td><td>Rabbit</td><td>Cell Signaling Technology</td><td>70076</td><td>IF</td></tr>
<tr><td rowspan="3">iNOS</td><td>1:100</td><td rowspan="3">Rabbit</td><td rowspan="2">HUABIO</td><td rowspan="2">HA722031</td><td>IF</td></tr>
<tr><td>1:200</td><td>IHC</td></tr>
<tr><td>1:1000</td><td>ABclonal Technology</td><td>A3774</td><td>Western blot</td></tr>
<tr><td rowspan="2">CD163</td><td>1:100</td><td rowspan="2">Rabbit</td><td rowspan="2">Abcam</td><td rowspan="2">ab182422</td><td>IF</td></tr>
<tr><td>1:200</td><td>IHC</td></tr>
<tr><td>γH2AX</td><td>1:500</td><td>Rabbit</td><td>HUABIO</td><td>ET1602-2</td><td>IF</td></tr>
<tr><td rowspan="4">CD44</td><td>1:5000</td><td rowspan="4">Rabbit</td><td rowspan="4">Abcam</td><td rowspan="4">ab189524</td><td>Western blot</td></tr>
<tr><td>1:500</td><td>IHC</td></tr>
<tr><td>1:200</td><td>IF</td></tr>
<tr><td>1:100</td><td>IP</td></tr>
<tr><td>β-Actin</td><td>1:2000</td><td>Rabbit</td><td>HUABIO</td><td>HA722023</td><td>Western blot</td></tr>
<tr><td rowspan="2">TNF-a</td><td>1:1000</td><td rowspan="2">Rabbit</td><td rowspan="2">Abmart</td><td rowspan="2">PY19810</td><td>Western blot</td></tr>
<tr><td>1:100</td><td>IF</td></tr>
<tr><td rowspan="2">IL-6</td><td>1:1000</td><td rowspan="2">Rabbit</td><td rowspan="2">Abmart</td><td rowspan="2">TD6087</td><td>Western blot</td></tr>
<tr><td>1:100</td><td>IF</td></tr>
<tr><td rowspan="2">IL-1β</td><td>1:1000</td><td rowspan="2">Rabbit</td><td rowspan="2">Abmart</td><td rowspan="2">P50520-1R1F</td><td>Western blot</td></tr>
<tr><td>1:100</td><td>IF</td></tr>
<tr><td rowspan="2">ELAVL1</td><td>1:200</td><td rowspan="2">Rabbit</td><td rowspan="2">Abmart</td><td rowspan="2">T57180F</td><td>IF</td></tr>
<tr><td>1:1000</td><td>Western blot</td></tr>
<tr><td>Flag-tag</td><td>1:2000</td><td>Mouse</td><td>Proteintech</td><td>66008-4-Ig</td><td>Western blot</td></tr>
<tr><td>GLS</td><td>1:1000</td><td>Rabbit</td><td>Proteintech</td><td>12855-1-AP</td><td>Western blot</td></tr>
<tr><td>Myc-tag</td><td>1:2000</td><td>Mouse</td><td>Proteintech</td><td>60003-2-Ig</td><td>Western blot</td></tr>
<tr><td>HA-tag</td><td>1:2000</td><td>Rabbit</td><td>Proteintech</td><td>51064-2-AP</td><td>Western blot</td></tr>
<tr><td>Goat anti Mouse IgG-HRP</td><td>1:10000</td><td>Goat</td><td>Beyotime</td><td>A0216</td><td>Western blot</td></tr>
<tr><td>Goat anti Rabbit IgG-HRP</td><td>1:10000</td><td>Goat</td><td>Beyotime</td><td>A0208</td><td>Western blot</td></tr>
</table>

Table S3. Antibodies for FACS.

Name	Fluorophore	Clone	Manufacture	Cat No.
Fixable Viability Stain 510	BV510	/	BD Pharmingen	564406
CD45	APC-CY7	30-F11	BD Pharmingen	557659
CD11B	FITC	M1/70	BD Pharmingen	557396
F4/80	BV421	T45-2342	BD Pharmingen	565411
CD86	PE	GL1	BD Pharmingen	553692
CD206	AF647	Y17-505	BD Pharmingen	568808
